# Psychosocial Risks in Informal Employment: A Scoping Review

**DOI:** 10.3390/ijerph23070846

**Published:** 2026-06-27

**Authors:** María F. Cuenca-Lozano, Gabriel A. Jaramillo-Ochoa

**Affiliations:** Departamento de Química y Producción, Universidad Técnica Particular de Loja, Loja 110107, Ecuador; gajaramillo10@utpl.edu.ec

**Keywords:** informal workers, psychosocial risks, mental health, precarious employment

## Abstract

**Highlights:**

**Public health relevance—How does this work relate to a public health issue?**

- Informal employment affects approximately 2 billion workers globally who lack structural regulation, social protection and basic labor rights. This review examines how these precarious working conditions operate as a social determinant of health and synthesizes evidence on their association with the psychological well-being of informal workers.
- The review links the unregulated nature of informal work to mental health outcomes reported across the included studies, showing that structural exposure to financial instability, long working hours and workplace violence is associated with increased symptoms of stress, anxiety and depression.

**Public health significance—Why is this work of significance to public health?**

- This scoping review consolidates fragmented evidence on psychosocial risks within informal labor markets, complementing the well-established literature on physical, chemical and ergonomic occupational hazards. It applies to the PRISMA-ScR protocol and MMAT to synthesize primary studies that document the public health relevance of informal work.
- The findings highlight intersectional vulnerabilities and report quantitative and qualitative evidence that marginalized subgroups—including female workers balancing unpaid caregiving and undocumented migrants exposed to occupational decline—face a disproportionate burden of mental health symptoms across the studies reviewed.

**Public health implications—What are the key implications or messages for practitioners, policymakers and/or researchers in public health?**

- For policymakers and practitioners, the review supports the extension of labor and social protection beyond formal employment boundaries—consistent with ILO Recommendation No. 204—as a structural lever to reduce psychosocial risk exposure among informal workers, including unpaid caregivers.
- For researchers, this work highlights the need to overcome primary data collection challenges in hidden populations by adopting comprehensive mixed-methods and longitudinal designs to map prevention factors and drive health equity.

**Abstract:**

**Background**: This review examines informal employment—defined here following ILO Recommendation No. 204 as remunerated and unremunerated jobs not effectively covered by formal arrangements—and its association with psychosocial risks. While particularly prevalent in low- and middle-income countries, informal employment is also a structural feature of high-income labor markets, affecting approximately 2 billion workers worldwide who lack regulation, security and social protection. These factors disproportionately affect vulnerable groups such as immigrants and women. **Method**: A scoping review was conducted across four databases (ScienceDirect, Scopus, Web of Science, PubMed) following the PRISMA-ScR extension. Eligibility was limited to primary studies on psychosocial risk factors in informal employment and their association with mental health (CRD420261280859) Prior systematic reviews were excluded from the evidence units and used only as back-ground literature. **Results**: The search yielded 257 initial articles, reduced after applying the eligibility criteria and after excluding prior systematic reviews and one out-of-scope study, to a final set of 12 primary studies. The review found a consistent association between informal employment and increased symptoms of stress, depression and anxiety, especially among women and undocumented immigrants. **Conclusions**: The findings support the extension of labor and social protection to informal workers, including unpaid caregivers, to reduce the adverse mental health effects of precarious employment. Socioeconomic exclusion, lack of labor rights and occupational decline increase vulnerability, underscoring the importance of addressing structural inequalities.

## 1. Introduction

This review addresses two concepts that lack a uniform definition in academic literature. Informal employment is understood, in line with International Labor Organization (ILO) Recommendation No. 204 [1] and the resolutions of the 17th [2] and 20th [3] International Conferences of Labour Statisticians, as remunerated and unremunerated jobs that are not effectively covered by formal arrangements, regardless of whether they take place in informal economic units, formal enterprises or households. It encompasses own-account workers, contributing family workers, informal employees and workers in non-regulated forms of employment. Informal employment is distinct from undeclared work (lawful activity deliberately concealed from authorities) and from gray employment (partially declared employment that complies with some, but not all, regulatory obligations). Following the same recommendation, extending labor and social protection to informal workers is a central policy lever for advancing decent work.

Psychosocial risks are defined, following the joint ILO/WHO framework [4] and EU-OSHA [5], as those aspects of the design, organization and management of work—together with their social and environmental context—that have the potential to cause psychological, physical or social harm. In this review, the term refers to exposures (e.g., excessive hours, job insecurity, harassment, work–family conflict, low social support), while mental health symptoms are treated as outcomes. The terms “occupational decline” (downward mismatch between a worker’s prior occupation and the occupation accessed, with loss of skill use and income) and “occupational/professional degradation” (broader sociological deterioration of working conditions, autonomy and recognition over time) are used consistently throughout.

Informal workers are mostly from the working class in low- and middle-income countries; it is estimated that there are 2 billion “informal” workers without social protection [6]. In Latin America and the Caribbean, approximately 50% of the workforce is informally employed under the ILO definition adopted above. These jobs are characterized by limited or absent legal coverage, low wages, insecure tenure, and frequent temporary, part-time or short-term arrangements [6,7,8].

Informal employment is considered a social and health determinant; workers are vulnerable to health risks and socioeconomic exclusions, poor working conditions and the general experience of precarious employment, which is associated, according to some authors, with poor health, increased risk of injuries, cardiovascular disorders, and decreased well-being of individuals and their families, and, in general, this is associated with health affectations. It was analyzed that exposure to chemicals and other hazardous substances; loud noise; heavy lifting; lack of breaks at work; and long working hours, without the necessary training, affect the health of informal workers. By denying workers fundamental principles and rights, informal employment jeopardizes life, health, liberty, security and human dignity [8,9].

Regarding the health of informal workers, studies are currently focused on the analysis of mental health in this group of workers, that is, those who meet the characteristics. Mental health has a significant impact on all aspects of people’s lives, and in the case of informal workers, due to precarious working conditions, there is an association with poor mental health and, therefore, the presence of depressive symptoms. It is indicated that undocumented immigrant workers are more exposed to risk factors for mental health due to professional degradation [7,10,11].

The problem highlights the need to analyze psychosocial risk factors in informal workers. These risk factors highlight some effects such as social disconnection, social isolation and/or loneliness, as well as the cumulative effect of multiple components of long-term social stress. Studies record effects of informal work such as episodes of anger, anxiety, depression, mental anguish and night terrors. Research determines that these effects are a consequence of invisibility, lack of rights, and unfair power relations, including disrespect and exploitation; violence is mentioned as an aggravating factor and lack of work breaks [9,12].

At the same time, another aspect of the problem has been identified, namely gaps in the amount of evidence and theoretical knowledge available to explain and understand the relationship between informal employment and psychosocial effects. There is a notable dispersion and fragmentation of evidence, with a predominantly limited focus on the relationship between specific psychosocial risks or the priority given to the physical, chemical, and ergonomic risks of informal employment. The literature also shows contradictory results and insufficient nuances, especially in the association between informal employment and mental health. Based on the methodological challenges inherent to primary data collection, such as the difficulty in obtaining information directly from these workers who have irregular schedules or unregistered contracts or due to cultural or language differences, and the scarcity of secondary sources, i.e., lack of official reports and statistics, it is proposed to establish a precise link between mental health variables and specific groups of informal workers. It should be emphasized that the study of occupational psychosocial risks is an established field; the specific gap addressed here is the absence of an integrated synthesis focused on informal workers as defined by ILO R204, where prior reviews exist only for adjacent topics (precarious employment, migrant labor, informal caregiving).

These shortcomings are considered to require a comprehensive synthesis of information in order to integrate the results and establish reliable conclusions [9,13,14,15]. In summary, this scoping review aims to provide information on informal employment and its relationship with psychosocial risk factors. Generating and synthesizing scientific evidence is considered essential as a contribution to risk management systems and decision-making worldwide. This information also helps to reduce gaps in existing data.

## 2. Material and Methods

The process of formulating the research question should include four fundamental elements: the participants, the exposures compared, their outcome and the design of the studies to address the problem or objective sought by the review. The information that can be obtained from the different psychosocial risk factors to which informal workers are exposed will serve as a basis for answering the research question posed and will support future research. It is necessary to mention that the research will provide a starting point on the research questions posed, with the purpose of providing further discussion on the topic. Therefore, the research question posed is the following:

RQ1. What are the psychosocial risk factors to which informal workers are exposed?

To deepen the scoping literature review, the following secondary research questions were specified:

RQ1a. Which working-condition characteristics of informal employment are most consistently associated with psychosocial risk exposure?

RQ1b. Which external (extra-occupational) and individual factors moderate or aggravate psychosocial risk in informal workers?

### 2.1. Inclusion and Exclusion Criteria

To answer the research question, inclusion and exclusion criteria were used according to PICO (patient/population, intervention, comparison, and outcome) and other criteria that were considered important, as shown in Table 1. Based on these criteria, databases were established in which information related to occupational health and safety, specifically psychosocial risk factors, was identified. The databases used were ScienceDirect, Scopus, Web of Science, and PubMed.

In each of the scientific bases, the bibliographic search was performed using keywords related to the research question posed. The search strategy combined three concept blocks: (i) informal employment (informal worker* OR informal employment OR informal economy OR precarious work* OR own-account worker* OR undocumented worker*); (ii) psychosocial exposures (psychosocial risk* OR psychosocial factor* OR work organization OR job strain OR job demand* OR work-family conflict OR workplace violence OR harassment); and (iii) mental health outcomes (mental health OR depression OR anxiety OR stress OR burnout OR psychological distress). Boolean operators and database-specific syntax are reported in full in File S3. Only scientific articles published from 2019 onwards were considered in order to capture evidence concurrent with the COVID-19 period. Setting 2019 as the lower bound of the search captures the pandemic period and allows COVID-19-related findings reported by the included studies to be synthesized within the review (see results Section 3).

### 2.2. Execution of the PRISMA-ScR Protocol

The research question marks the development of the scoping review and allows the development of the PRISMA-ScR protocol by materializing its stages, screening the articles by title and abstract, and defining the suitability of the articles by their content and the number of quantitative and qualitative studies. One of the difficulties in a systematic review is to find quality studies that contribute to the research. Therefore, it is important to use a checklist to evaluate the quality of each of the studies found in the search. This will allow the researcher to identify possible biases [16,17,18]. The reporting follows the PRISMA Extension for Scoping Reviews [19], and the completed checklist is provided as Supplementary Material.

**Figure 1:**
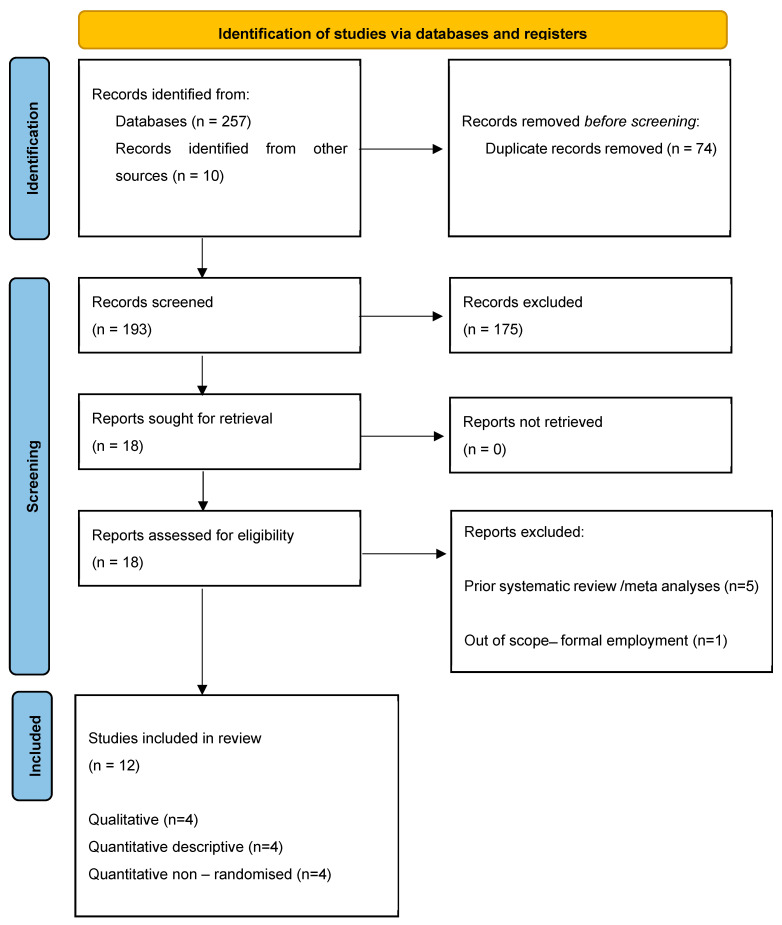
Review protocol with the methodological procedures adopted. Note: Figure shows the structure for identifying studies through databases and registries according to the PRISMA ScR 2018 flow diagram for scoping reviews.

After the execution of the PRISMA protocol, it is important to synthesize the results obtained. The interpretation of the evidence obtained provides relevance to the search, which allows the knowledge to be firm and the results to be presented in such a way that the strength of the evidence supporting each outcome can be evaluated [20,21]. Figure 1 shows the protocol execution.

The identification phase shows the number of records found, which represents the basis of the review. The screening and suitability process are seen as an adequate filter towards the final identification of the number of included studies. In relation to those excluded, the analysis factors respond to articles outside the time period, ambiguous constructs and imprecise or duplicate titles.

## 3. Results Description

This section details all the information obtained from the selected studies. As can be seen in Table 2, after performing the search process, applying the selected criteria, a total of 267 articles were found in the first stage. Subsequently, in stage two, the search was reduced to 193 articles and to a final base of 12 primary studies (after excluding 5 prior systematic reviews/meta-analyses and 1 out-of-scope study).

After the search supported by the phases described above, the results are obtained. Table 3 below presents the characteristics of the 12 included primary studies, charting for each one the country and income setting (HIC/LMIC), study design, sample size, operational definition of informal work, comparison group(s), instruments used to assess psychosocial exposures and mental health outcomes, key findings and statistical indicators.

The structure of Table 3 allows for a comparative evaluation of the evidence. The information highlights a heterogeneity of designs across the included primary studies, encompassing prospective cohort studies, cross-sectional surveys with logistic and Poisson regression, latent-class analysis, and qualitative ethnographic and interview-based approaches. From a technical perspective, internationally validated psychometric instruments such as the Patient Health Questionnaire (PHQ-9), the Center for Epidemiologic Studies Depression Scale (CES-D 10), the General Health Questionnaire (GHQ-12), and the Job Content Questionnaire (JCQ) are reported, ensuring the reliability and validity of the mental health outcomes analyzed. Quantitative studies report Prevalence Ratios (PR) and Odds Ratios (OR) with their respective 95% confidence intervals as indicators of statistical robustness.

The Mixed Methods Appraisal Tool (MMAT) was used for the critical appraisal of the 12 included primary studies, evaluating qualitative, quantitative non-randomized, and quantitative descriptive designs The detailed criteria for each design category (qualitative 1.1–1.5; quantitative non-randomized 3.1–3.5; quantitative descriptive 4.1–4.5) are reported in File S2 and in the last column of Table 4. Y indicates that the criterion was met; N that it was not met; CT that it could not be told from the available information; and NR that the original study did not report the information required to judge the criterion.

### COVID-19-Related Findings

Two of the included studies [11,23] explicitly examined psychosocial exposures during the COVID-19 period.

Across these studies, the pandemic was associated with intensified work–family conflict among unpaid female caregivers, increased job insecurity among informal migrant workers, and amplified social isolation among undocumented day laborers. These findings are integrated into Section 4 alongside the broader evidence base, while acknowledging that the limited number of pandemic-specific studies precludes formal subgroup analysis.

## 4. Discussion

Synthesizing the existing scientific evidence on psychosocial risk factors associated with informal work was the objective of this research. The informal worker sector is a growing sector worldwide, including high-income countries [9]. The innate characteristics of informal employment demand a thorough understanding of these risks as an input to management systems and decision-making processes aimed at protecting workers. The review also sought to show the various methodological approaches used in this area of research and to identify gaps in the current theoretical understanding; thus, the notable dispersion and fragmentation of evidence regarding psychosocial risks in informal employment was identified as part of the problem. While there is a predominant focus on physical, chemical and ergonomic hazards, the psychosocial dimension has received less concentrated attention, and there is a need for more comprehensive research approaches to meet the needs of this context. Faced with the need for comprehensive syntheses, findings have been grouped according to psychosocial factors related to informal employment in order to identify reliable patterns, which are discussed below:

### 4.1. Working Conditions

In answer to RQ1a, the working-condition characteristics most consistently associated with psychosocial risk exposure across the included studies are low wages, lack of labor rights and social-protection coverage, long or unpredictable hours, exposure to violence and harassment, and weak interpersonal relationships at work. The remainder of this section presents the supporting evidence and the boundary conditions identified.

These patterns are consistent with two well-established occupational health frameworks. The demand–control–support and effort–reward imbalance models predict that jobs combining high demands with low control and low reward elevate the risk of common mental disorders [29,30]. Informal employment concentrates precisely this configuration: long or unpredictable hours and workplace violence on the demand side, and absent labor rights, low wages and weak social support on the reward side.

The magnitude of the association varies with exposure intensity. The 27% excess prevalence of major depressive symptoms reported for informal workers as a whole (PR 1.27; 95% CI 1.00–1.62) rises to an OR of 3.47 (95% CI 1.46–8.28) among Hispanic au pairs working >40 h per week and to an OR of 4.95 (95% CI 2.16–9.75) when physical violence is present. This gradient suggests that informality alone is not the active mechanism; what matters is the underlying configuration of demands, control and rewards in each occupational niche [31].

The review highlights poor working conditions as a central theme consistently linked to adverse psychosocial outcomes in informal employment. Studies show that low wages, lack of labor rights, poor interpersonal relationships, and lack of social security contribute significantly to mental health problems. Among the included primary studies, Ref. [7] found a 27% (Prevalence Ratio [PR]: 1.27; 95% CI: 1.00, 1. 62) higher association of informal employment with major depressive symptoms compared to formal workers, with this association persisting regardless of gender, with female informal workers showing a higher prevalence ratio (PR: 1.36; 95% CI: 1.06, 1.74) and male informal workers showing a similar trend (PR: 1.22; 95% CI: 0.90, 1.65). These data point to the systemic nature of the problem, which affects both genders in informal settings. As background, prior systematic reviews on adjacent populations [15] have likewise reported elevated depressive-symptom prevalence among paid domestic workers, providing converging context for the patterns identified in the primary studies synthesized here.

For immigrant workers, the included primary studies consistently link precarious employment with mental health problems. Qualitative studies reinforce this finding, framing precarious employment as a key driver of these mental health challenges. Ref. [8] highlighted this by demonstrating that levels of job insecurity, health status and well-being varied with the immigration status of informal workers in New York City; the study addresses the analysis of policies that promote health equity. Other studies propose the analysis of other factors; for example, Ref. [22] through ethnographic research, revealed how unstable working conditions, demotivation, lack of social security and vulnerability profoundly impact mental health and exacerbate occupational risks among informal workers. These observations are consistent with the framing offered by prior reviews on precarious and informal employment [32,33], cited here as background literature, not as evidence units of this scoping review.

Women in informal employment and the challenges they face are also discussed in this review. Studies examining migrant women workers [23] identified precarious conditions, ethnic and gender discrimination, and family rejection of women workers as factors affecting well-being, in contrast to improvements in working conditions, such as skills development and quality working time, which demonstrated improved mental health and reduced anxiety, social problems, and risk of depression in these workers. Longitudinal studies, although limited, provide critical information, for example, Ref. [25] observed that Hispanic au pairs in Germany who worked more than 40 h per week were more than three times more likely to suffer from depression (OR: 3.47; 95% CI: 1.46–8.28). Exposure to physical violence increased this risk almost fivefold (OR: 4.95; 95% CI: 2.16–9.75), and poor schedule adaptation to social and family commitments doubled the risk (OR: 2.24; 95% CI: 0.95–5.28). The data underscore the vulnerability faced by informal workers due to long hours, violence, and work–life imbalance.

This review also pools information on informal caregivers. A large-scale cohort study by [27] did not find a direct association between informal caregiving or high job strain and the risk of developing pathologies such as type 2 diabetes. However, it highlights that low social support at work is identified as a risk factor for type 2 diabetes (OR: 1.18, 95% CI: 1.02–1.37), highlighting the importance of social support groups regardless of work status, i.e., formal or informal. Beyond the primary studies synthesized above, a prior meta-analysis on intervention-based research [34] suggests that e-health approaches may benefit mental health in informal caregivers of cancer patients; this work is cited here as background literature only and not as evidence directly synthesized in this scoping review.

To close this section, the comparison between formal and informal workers does not point in a single direction. [28] reported greater upper-extremity musculoskeletal pain among formal workers exposed to high demands (PR = 1.69; 95% CI: 1.46–1.96) than among their informal counterparts (PR = 1.40; 95% CI: 1.30–1.51). This counterintuitive finding suggests that psychosocial-risk patterns are not uniformly worse in informal employment and that exposure profiles must be assessed by occupation and context rather than by formality alone.

### 4.2. Individual Characteristics and Extra-Occupational Factors

In answer to RQ1b, the external (extra-occupational) and individual factors that most consistently moderate or aggravate psychosocial risk among informal workers are gender and care responsibilities, migration and legal status, occupational decline, social isolation and exclusion, and the macro-shock represented by the COVID-19 pandemic. The supporting evidence is summarized below.

The factors identified in RQ1b are best read as intersecting axes of vulnerability rather than as independent variables. Gender, migration status, occupational decline and social exclusion tend to co-occur in specific subgroups of informal workers, and their combined effect is not captured by simple formal vs. informal comparisons [35]. The synthesized studies illustrate this in undocumented immigrant men, where occupational decline reaches 66.63% and is associated with odds ratios of 1.73 to 2.66 for mental disorders, and in migrant women workers, where ethnic and gender discrimination act simultaneously with care responsibilities.

From a social-determinants perspective, these clustered exposures sustain chronic stress responses and amplify the impact of any single occupational stressor [31]. The COVID-19 period operated as a macro-shock that intensified pre-existing factors—particularly work–family conflict among unpaid female caregivers and job insecurity among informal migrant workers—rather than introducing new ones. This reading explains why single-factor interventions tend to yield limited mental health benefits in informal-worker populations.

In this section, individual experiences and circumstances external to work, known as extra-occupational factors, influence the mental health of informal workers. The COVID-19 period—captured by two of the included primary studies [11,23]—coincided with intensified work–family conflict among unpaid female caregivers and amplified job insecurity among informal migrant workers, underscoring the disproportionate burden of care that generally falls on women. A prior systematic review of high-income OECD countries [36] provides converging background evidence on the negative association between unpaid caregiving and mental health among working-age adults; it is cited here as background literature only. Work–family conflict emerged as a significant stressor affecting mental health [10]. Other stressors identified included demands on work time, low rewards, threats at work (such as sexual harassment), and low social support. The study’s robust statistical analysis (KMO of 0.945, Bartlett’s test *p* < 0.05) supports the strong relationship between these variables and mental health outcomes.

This review made evident the unique vulnerabilities of undocumented immigrant workers. Ref. [11] found a prevalence of mental illness of 5.58% in this group and highlighted the impact of occupational decline, with 66.63% experiencing such a decline. Logistic regression models indicated that undocumented immigrants who experience occupational decline are at significantly increased risk for mental disorders, with odds ratios ranging from 1.729 (95% CI: 1.071–2.793) when controlling for individual characteristics to 2.659 (CI: 1.342–5.271) with all controls; this highlights the profound psychological cost of precarious legal status and devalued work. Social isolation and exclusion, particularly for undocumented immigrant men, were identified as critical factors exacerbating mental health problems [12]. The ethnographic study with Latino migrant day laborers revealed experiences of racism, structural vulnerability, dehumanization and substance use, all of which contribute to significant health deterioration, further complicated by the lack of Latino migrant communities or social infrastructure in certain cities, which further exacerbates this isolation.

### 4.3. Informal Employment Across Income Settings

The included evidence spans both low- and middle-income (LMIC) and high-income (HIC) contexts. In LMICs, informality is typically the dominant labor-market arrangement and overlaps with limited formal social protection; in HICs, it concentrates in gig work, undocumented migration and unpaid caregiving. The mental health implications of informal work differ accordingly. In some HIC contexts, informal arrangements may offer flexibility that, when paired with adequate safety and compensation, can be associated with neutral or positive well-being outcomes. The synthesis presented in Section 4.1 and Section 4.2 should be read with this heterogeneity in mind.

### 4.4. Policy Implications for Unpaid Informal Caregivers

Unpaid informal caregivers—predominantly women—bear a measurable mental health burden that policy responses for paid informal workers cannot, on their own, address. Systematic-review evidence shows that informal caregiving is associated with depressive symptoms and reduced psychological well-being, with effect sizes that grow with care intensity and concentrate among female and co-residing caregivers [37,38]. European population data converge on the same pattern, and the gap widens where formal long-term care provision is weaker [39].

### 4.5. Future Research Agenda

The synthesis surfaced gaps that translate into five priority lines for future research. (i) Standardized measurement of informality and of psychosocial exposures across LMIC contexts, so that comparisons across studies become possible. (ii) Longitudinal designs capable of distinguishing accumulation effects from reverse causation between informal work and mental health, with outcomes assessed at multiple time points. (iii) Intersectional analyses of gender, migration status, occupation and care work—moving beyond aggregated comparisons of formal vs. informal—to identify the subgroups carrying the highest burden.(iv) Intervention research, including pilots that extend social protection to informal workers (ILO R204) and workplace-based mental health interventions adapted to informal settings (e.g., for domestic workers, day laborers, gig-economy workers). (v) Methodological innovations for reaching hidden populations, including respondent-driven sampling, community-based participatory research and digital ethnography. For each of these lines, future studies should specify the substantive research question, the target population and the design implications to produce evidence that is operationally useful for policy.

Two policy logics must therefore run in parallel. For paid informal workers, the dominant lever remains the extension of labor standards and social insurance, in line with ILO Recommendation No. 204. For unpaid caregivers—outside any contractual employment relationship—the relevant frame is the care economy.

The ILO “5R” framework structures this second response: recognize unpaid care work in statistics and social-protection eligibility; reduce its volume through accessible care services; redistribute it between women, men and the state; reward paid care work with decent wages; and represent caregivers in policy design [40]. In practice this means respite services, mental health support, caregiver allowances linked to pension credits and gender-aware program design. Treating the two logics in isolation risks leaving the largest subgroup of informal workers—unpaid female caregivers—outside the protective reach of both occupational health and welfare policy.

## 5. Conclusions

In direct answer to RQ1, the psychosocial risk factors most consistently identified across the included studies fall into two clusters. Working-condition factors include low wages, lack of labor rights and social protection, long or unpredictable hours, workplace violence and harassment, and weak interpersonal relationships. Individual and extra-occupational factors include work–family conflict, gendered care responsibilities, migration and legal status, occupational decline, social isolation, and exposure to the COVID-19 macro-shock. Both clusters are interrelated and are associated with elevated symptoms of depression, anxiety and chronic stress in informal workers.

The main objective of this scoping review was to synthesize the scientific evidence on psychosocial risk factors associated with informal work, showing the complex and diverse nature of these risks, which are generally overlooked, prioritizing predominantly physical, chemical and ergonomic risks. Due to the growing prevalence of informal employment globally, even in high-income countries, the need to generate information to understand these risks to enrich the information bases that contribute to management systems and policy decisions aimed at protecting workers is highlighted. A central finding is the precariousness of working conditions in the informal sector, i.e., working conditions in this sector are unstable and are consistently linked to adverse psychosocial outcomes or impairments of mental and emotional well-being. The review identified that low wages, lack of labor rights, poor interpersonal relationships and lack of social security contribute significantly to mental health problems, including depression and anxiety. Domestic workers and migrant workers stand out as vulnerable groups, with studies showing a high prevalence of depressive symptoms and influence on mental health problems complicated by poverty, intersectional discrimination and job insecurity. In addition, long working hours, violence and work–life imbalance aggravate the vulnerability of this group of workers.

In addition to working conditions, factors such as individual and extra-occupational characteristics play an important role in the mental health of informal workers; work–family conflict, insufficient pay, threats in the work environment such as sexual harassment, and low social support are significant aggravating factors in this problem. In the case of undocumented immigrants, factors such as occupational decline, social isolation, exclusion and racism add to the significant deterioration of mental health, showing the profound psychological consequences of informal working conditions. Efforts to generate a synthesis of valid information through this review highlight issues such as gaps in the literature, a critical need for future research towards more comprehensive frameworks to specifically understand the relationship between informal work and psychosocial effects, and the use of stronger methodologies such as longitudinal designs or mixed methods that capture the complex interaction between individuals and society. Exploring prevention factors specific to informal worker populations and developing effective, ethical and efficient methods for primary data collection are considered necessary actions in response to the problem. In addition, prioritizing the understanding of psychosocial risks to generate accurate information to drive the development of policies aimed at risk prevention and the promotion of health equity.

Limitations. This review has three principal limitations. First, the search was restricted to English-language publications, which likely under-represents LMIC contexts where research is published primarily in Spanish, Portuguese or Chinese; multilingual evidence syntheses are recommended. Second, the review’s scoping nature precludes formal meta-analytic pooling. Third, the heterogeneity of operational definitions of informality across the included studies limits direct comparability of effect sizes.

## Figures and Tables

**Table 1:** Inclusion and exclusion criteria according to PICO (Patient/Population, Intervention, Comparison and Outcome).

	Inclusion	Exclusion
Population	Workers in informal employment Domestic workers Digital platform workers without contract Street vendors	Workers with formal employment Workers without a history of informal employment
Exposure/Intervention	Studies examining the experience or characteristics of informal employment	Specific interventions to improve mental health Interventions to improve psychosocial conditions in informal employment
Comparison	Psychosocial effects/mental health between informal and formal work, among subgroups of informal workers or among informal workers without a comparison group	Without relevant comparison groups Without a clear description of psychosocial effects within the informal population
Outcome	Depression, anxiety, stress, burnout, mental health Autonomy, control over work, social support, work–life balance, job precarity	Physical, chemical, ergonomic risks Work accidents
Study characteristics	Observational, qualitative studies and systematic reviews in English from 2019 onward	Opinion pieces, editorials, letters to the editor, individual case reports in a language other than English and before 2019

Note on inclusion of prior reviews: Prior systematic reviews and meta-analyses on adjacent topics were excluded from the core evidence synthesis to avoid double-counting of primary studies. They are cited in the Discussion only as background literature, with sources clearly distinguished from the primary studies that constitute the evidence units of this review.

**Table 2:** Number of articles filtered at each step.

Selections of Studies	Articles
Identification	267
Screening	193
Eligibility	18
Inclusion	12

**Table 3:** Overview of the literature used.

Author/Year	Country/Setting	Study Design	Sample Size & Population	Operational Definition of Informal Work	Comparison Group(s)	Pyschosocial Exposure Instrument	Mental Health Outcome Instrument	Key Findings	Statistical Indicators
[7]	11 cities, Latin America & Caribbean (LMIC)	Cross-sectional	*n* ≈ 7000 adults, formal vs. informal employment subsamples in 11 LAC cities	Workers not contributing to the social security/pension system (national labor-force surveys)	Formal vs. informal workers; subgroups by sex	Self-reported employment status; demographic & job items	Short Depression Scale (CES-D 10-item adaptation)	Informal employment associated with a higher prevalence of major depressive symptoms; persists by sex	PR 1.27 (95% CI 1.00–1.62); women PR 1.36 (1.06–1.74); men PR 1.22 (0.90–1.65)
[11]	Italy (HIC)—undocumented migrants	Cross-sectional + logistic regression	*n* = 994 undocumented migrants seeking care at NAGA clinic, Milan	Undocumented migrant workers without legal residence/labor contract	Workers with vs. without occupational decline	Self-reported occupational history; legal status & decline classification	Clinical diagnosis (ICD-10) by NAGA clinicians	5.58% mental-illness prevalence; 66.63% experienced occupational decline; decline strongly associated with mental-disorder risk	OR 1.729 (95% CI 1.071–2.793); fully adjusted OR 2.659 (1.342–5.271)
[8]	New York City, USA (HIC)	Qualitative—semi-structured interviews	*n* = 45 precariously employed workers, mixed immigration status	Non-standard/precarious employment (no contract, no benefits, gig work)	Subgroups by immigration status	Interview guide on job conditions, insecurity, well-being	Self-reported well-being narratives	Job insecurity, health and well-being vary by immigration status; calls for equity-oriented policy	NR (qualitative)
[12]	Baltimore & Washington DC, USA (HIC)	Qualitative ethnographic—8 focus groups	Latino migrant day laborers; 8 focus groups	Day labor informal hiring (esquineros), no employment contract	Within-group narrative analysis	Ethnographic field notes; focus-group guide on isolation, dehumanization	Self-reported mental health and substance-use narratives	Structural social exclusion → racism, dehumanization, substance use; high isolation	NR (qualitative)
[22]	Colombia (LMIC)—Venezuelan immigrants	Qualitative—ethnographic, Atlas.Ti 8.0	Venezuelan immigrant population in Colombia	Precarious migrant employment, mostly informal	Within-group thematic analysis	Semi-structured interviews; ethnographic notes	Self-reported mental health and well-being narratives	Employment instability, demotivation, lack of social security and vulnerability impact mental health and exacerbate occupational risks	NR (qualitative)
[23]	United Kingdom (HIC)	Quantitative longitudinal—panel data	UK Household Longitudinal Study microdata linked with European Working Conditions Survey (EWCS)	Precarious working conditions (low pay, low control, short tenure)—overlap with informal arrangements for migrant women	Changes within individuals over time; men vs. women	EWCS items (working conditions: skills, time, intensity)	GHQ-12 (General Health Questionnaire)	Improved working conditions linked to better mental health and lower anxiety/depression risk, especially for women	Statistically significant correlations reported; effect sizes vary by domain
[24]	Turkey (LMIC)—domestic and informal workers	Qualitative—semi-structured interviews	Informal women workers; small-sample qualitative design	Informal own-account/domestic work, no contractual coverage	Within-group thematic analysis	Interview guide on working conditions, family support, social life	Self-reported well-being narratives	Family support and positive social life buffer the psychosocial burden of poor working conditions	NR (qualitative)
[10]	China (LMIC)—multi-organization online survey	Cross-sectional online survey	*n* = 336 workers from various organizations	Workers in non-standard/informal arrangements (mix of own-account, platform, casual)	Stressor categories	Stressor scale (work-time demands, low rewards, threats, work–family conflict, low social support)	Mental health outcome scale (self-report)	Work–family conflict and threats at work strongly associated with adverse mental health	KMO 0.945; Bartlett *p* < 0.05; significant associations across stressors
[25]	Germany (HIC)—Hispanic au pairs	Prospective cohort, longitudinal	*n* = 119 Spanish-speaking au pairs in Germany; multiple measurement intervals	Au-pair work—informal household employment with limited legal coverage	Au pairs working > 40 h/week vs. ≤40 h/week; exposure to violence yes/no	Survey on working hours, social/family schedule, exposure to violence	PHQ-9 (Patient Health Questionnaire-9)	Working > 40 h/week tripled depression risk; physical violence increased risk ~5×; poor schedule adaptation doubled risk	>40 h/week OR 3.47 (1.46–8.28); violence OR 4.95 (2.16–9.75); schedule mismatch OR 2.24 (0.95–5.28)
[26]	USA (HIC)	Cross-sectional—latent class analysis	Nationally representative US workforce sample	Non-standard employment (contractor, temp, on-call, gig)	Standard vs. non-standard employment classes	Composite job-quality index (income, schedule, benefits, autonomy)	Self-reported physical and mental health items; occupational injury	Lower job quality associated with worse mental health and higher occupational-injury prevalence	Statistically significant ORs across job-quality classes (95% CI reported in source)
[27]	Denmark (HIC)	Prospective cohort	*n* ≈ 4600 working-age adults followed for incidence of T2D and CVD	Informal caregiving alongside paid employment; job strain measured by JCQ	High vs. low job strain; informal caregiving yes/no	Job Content Questionnaire (JCQ)—job-strain & social-support items	Registry-confirmed incidence of T2D	Informal caregiving and high job strain not directly associated with T2D risk; low social support at work increases T2D risk	Low social support OR 1.18 (95% CI 1.02–1.37)
[28]	Central America (LMIC)—6 countries	Cross-sectional—Poisson regression	*n* ≈ 12,000 workers from the I Central American Working Conditions Survey	Self-reported informal employment (no written contract, no social security contribution)	Formal vs. informal workers	Psychosocial work-risk items derived from COPSOQ/JCQ adapted to the survey	Self-reported upper-extremity musculoskeletal pain	High psychosocial demands → greater upper-extremity pain in formal workers than informal workers (counterintuitive direction discussed in Section 4.1)	Formal PR 1.69 (1.46–1.96); informal PR 1.40 (1.30–1.51)

Note. HIC = high-income country; LMIC = low- and middle-income country (World Bank classification). NR = not reported in the original study.

**Table 4:** Methodological quality analysis.

No.	Author (Year)	Category/Design	S1	S2	C1	C2	C3	C4	C5	Quality/Validation	MMAT Category & Criterion Key
1	[7]	Quant. Descriptive	Y	Y	Y	Y	Y	NR	Y	80% (****)	4.1 Sampling strategy; 4.2 Representative sample; 4.3 Appropriate measurements; 4.4 Low non-response bias; 4.5 Appropriate statistical analysis
2	[11]	Quant. No Rand.	Y	Y	Y	Y	Y	Y	Y	100% (*****)	3.1 Participants representative; 3.2 Appropriate measurements; 3.3 Complete outcome data; 3.4 Confounders accounted for; 3.5 Intervention/exposure as intended
3	[8]	Qualitative	Y	Y	Y	Y	Y	Y	Y	100% (*****)	1.1 Qualitative approach appropriate; 1.2 Data collection adequate; 1.3 Findings derived from data; 1.4 Interpretation substantiated; 1.5 Coherence
4	[12]	Qualitative	Y	Y	Y	Y	Y	Y	Y	100% (*****)	1.1–1.5 (see qualitative key above)
5	[22]	Qualitative	Y	Y	Y	Y	Y	Y	Y	100% (*****)	1.1–1.5 (see qualitative key)
6	[23]	Quant. No Rand.	Y	Y	Y	Y	Y	Y	Y	100% (*****)	3.1–3.5 (see non-randomized key)
7	[24]	Qualitative	Y	Y	Y	Y	Y	Y	Y	100% (*****)	1.1–1.5 (see qualitative key)
8	[10]	Quant. Descriptive	Y	Y	Y	Y	Y	NR	Y	80% (****)	4.1–4.5 (see descriptive key)
9	[25]	Quant. No Rand.	Y	Y	Y	Y	Y	Y	Y	100% (*****)	3.1–3.5 (see non-randomized key)
10	[26]	Quant. Descriptive	Y	Y	Y	Y	Y	Y	Y	100% (*****)	4.1–4.5 (see descriptive key)
11	[27]	Quant. No Rand.	Y	Y	Y	Y	Y	Y	Y	100% (*****)	3.1–3.5 (see non-randomized key)
12	[28]	Quant. Descriptive	Y	Y	Y	Y	Y	Y	Y	100% (*****)	4.1–4.5 (see descriptive key)

NR: Not reported. Information not reported in the original study to evaluate the criterion. Following the guidelines of the Mixed Methods Appraisal Tool 2018 manual (File S3), in the absence of clear data on non-response bias or participation rates, the rating ‘Not reported’ was assigned, which is interpreted as a criterion not met for the calculation of the overall quality percentage. (*****): 100% of the quality criteria; (****): 80% of the quality criteria; As additional information, it should be noted that editorial validation and impact factor validation were carried out for systematic reviews as an indicator of methodological rigor. The use of the systematic reviews considered for this article is justified because they respond to the research question and the gap identified in the existing literature.

## Data Availability

No new data were created or analyzed in this study..

## Supplementary Materials

The following supporting information can be downloaded at: https://www.mdpi.com/article/10.3390/ijerph23070846/s1, File S1: PRISMA 2020 Checklist; File S2: MMAT Analysis Criteria; File S3: Search Strategy.
