# Supplementary material for: Psychosocial Risks in Informal Employment: A Scoping Review
Source: Int J Environ Res Public Health. 2026 Jun 27;23(7):846. doi: 10.3390/ijerph23070846 (PMC13411353; doi:10.3390/ijerph23070846)
Supplement: Supplementary file 1 [file ijerph-23-00846-s001.zip › ijerph-4355514-supplementary.pdf]

File S1. Preferred Reporting Items for Systematic reviews and Meta-Analyses extension for Scoping Reviews (PRISMA-ScR) Checklist

SECTION	ITEM	PRISMA-ScR CHECKLIST ITEM	REPORTED ON PAGE #
**TITLE**			
Title	1	Identify the report as a scoping review.	1
**ABSTRACT**			
Structured summary	2	Provide a structured summary that includes (as applicable): background, objectives, eligibility criteria, sources of evidence, charting methods, results, and conclusions that relate to the review questions and objectives.	2
**INTRODUCTION**			
Rationale	3	Describe the rationale for the review in the context of what is already known. Explain why the review questions/objectives lend themselves to a scoping review approach.	2-3; 5
Objectives	4	Provide an explicit statement of the questions and objectives being addressed with reference to their key elements (e.g., population or participants, concepts, and context) or other relevant key elements used to conceptualize the review questions and/or objectives.	4
**METHODS**			
Protocol and registration	5	Indicate whether a review protocol exists; state if and where it can be accessed (e.g., a Web address); and if available, provide registration information, including the registration number.	2
Eligibility criteria	6	Specify characteristics of the sources of evidence used as eligibility criteria (e.g., years considered, language, and publication status), and provide a rationale.	5; 15
Information sources*	7	Describe all information sources in the search (e.g., databases with dates of coverage and contact with authors to identify additional sources), as well as the date the most recent search was executed.	4
Search	8	Present the full electronic search strategy for at least 1 database, including any limits used, such that it could be repeated.	19

SECTION	ITEM	PRISMA-ScR CHECKLIST ITEM	REPORTED ON PAGE #
Selection of sources of evidence†	9	State the process for selecting sources of evidence (i.e., screening and eligibility) included in the scoping review.	6
Data charting process‡	10	Describe the methods of charting data from the included sources of evidence (e.g., calibrated forms or forms that have been tested by the team before their use, and whether data charting was done independently or in duplicate) and any processes for obtaining and confirming data from investigators.	10
Data items	11	List and define all variables for which data were sought and any assumptions and simplifications made.	7-9
Critical appraisal of individual sources of evidence§	12	If done, provide a rationale for conducting a critical appraisal of included sources of evidence; describe the methods used and how this information was used in any data synthesis (if appropriate).	10
Synthesis of results	13	Describe the methods of handling and summarizing the data that were charted.	5
**RESULTS**			
Selection of sources of evidence	14	Give numbers of sources of evidence screened, assessed for eligibility, and included in the review, with reasons for exclusions at each stage, ideally using a flow diagram.	6
Characteristics of sources of evidence	15	For each source of evidence, present characteristics for which data were charted and provide the citations.	7-9
Critical appraisal within sources of evidence	16	If done, present data on critical appraisal of included sources of evidence (see item 12).	10
Results of individual sources of evidence	17	For each included source of evidence, present the relevant data that were charted that relate to the review questions and objectives.	11-12
Synthesis of results	18	Summarize and/or present the charting results as they relate to the review questions and objectives.	11-14
**DISCUSSION**			
Summary of evidence	19	Summarize the main results (including an overview of concepts, themes, and types of evidence available), link to the review questions	11-14

SECTION	ITEM	PRISMA-ScR CHECKLIST ITEM	REPORTED ON PAGE #
		and objectives, and consider the relevance to key groups.	
Limitations	20	Discuss the limitations of the scoping review process.	15
Conclusions	21	Provide a general interpretation of the results with respect to the review questions and objectives, as well as potential implications and/or next steps.	14-15
**FUNDING**			
Funding	22	Describe sources of funding for the included sources of evidence, as well as sources of funding for the scoping review. Describe the role of the funders of the scoping review.	15

JBI = Joanna Briggs Institute; PRISMA-ScR = Preferred Reporting Items for Systematic reviews and Meta-Analyses extension for Scoping Reviews.
* Where *sources of evidence* (see second footnote) are compiled from, such as bibliographic databases, social media platforms, and Web sites.
† A more inclusive/heterogeneous term used to account for the different types of evidence or data sources (e.g., quantitative and/or qualitative research, expert opinion, and policy documents) that may be eligible in a scoping review as opposed to only studies. This is not to be confused with *information sources* (see first footnote).
‡ The frameworks by Arksey and O'Malley (6) and Levac and colleagues (7) and the JBI guidance (4, 5) refer to the process of data extraction in a scoping review as data charting.
§ The process of systematically examining research evidence to assess its validity, results, and relevance before using it to inform a decision. This term is used for items 12 and 19 instead of "risk of bias" (which is more applicable to systematic reviews of interventions) to include and acknowledge the various sources of evidence that may be used in a scoping review (e.g., quantitative and/or qualitative research, expert opinion, and policy document).

From: Tricco AC, Lillie E, Zarin W, O'Brien KK, Colquhoun H, Levac D, et al. PRISMA Extension for Scoping Reviews (PRISMAScR): Checklist and Explanation. Ann Intern Med. 2018;169:467–473. doi: 10.7326/M18-0850.

Flie S2. MMAT Analysis, Criteria

Part I: Mixed Methods Appraisal Tool (MMAT), version 2018

**Category of study designs**	**Methodological quality criteria**	**Responses**			
		Yes	No	Can't tell	Comments
Screening questions (for all types)	S1. Are there clear research questions?				
	S2. Do the collected data allow to address the research questions?				
	Further appraisal may not be feasible or appropriate when the answer is 'No' or 'Can't tell' to one or both screening questions.				
1. Qualitative	1.1. Is the qualitative approach appropriate to answer the research question?				
	1.2. Are the qualitative data collection methods adequate to address the research question?				
	1.3. Are the findings adequately derived from the data?				
	1.4. Is the interpretation of results sufficiently substantiated by data?				
	1.5. Is there coherence between qualitative data sources, collection, analysis and interpretation?				
2. Quantitative randomized controlled trials	2.1. Is randomization appropriately performed?				
	2.2. Are the groups comparable at baseline?				
	2.3. Are there complete outcome data?				
	2.4. Are outcome assessors blinded to the intervention provided?				
	2.5 Did the participants adhere to the assigned intervention?				
3. Quantitative non-randomized	3.1. Are the participants representative of the target population?				
	3.2. Are measurements appropriate regarding both the outcome and intervention (or exposure)?				
	3.3. Are there complete outcome data?				
	3.4. Are the confounders accounted for in the design and analysis?				
	3.5. During the study period, is the intervention administered (or exposure occurred) as intended?				
4. Quantitative descriptive	4.1. Is the sampling strategy relevant to address the research question?				
	4.2. Is the sample representative of the target population?				
	4.3. Are the measurements appropriate?				
	4.4. Is the risk of nonresponse bias low?				
	4.5. Is the statistical analysis appropriate to answer the research question?				
5. Mixed methods	5.1. Is there an adequate rationale for using a mixed methods design to address the research question?				
	5.2. Are the different components of the study effectively integrated to answer the research question?				

Flie S3. Search strategy

Manuscript ID: ijerph-4355514 — Psychosocial Risks in Informal Employment: A Scoping Review.

This appendix documents the bibliographic search strategy used to identify primary studies for the present scoping review. The reporting follows the PRISMA-ScR guidance (Tricco et al., 2018), which acknowledges that scoping reviews typically employ a more flexible search strategy than systematic reviews, prioritising conceptual coverage of the topic over exhaustive sensitivity.

1 Conceptual structure of the search

The search combined three concepts, joined by the Boolean operator AND. Within each concept, the keyword listed below was used as the entry term.

Concept	Keyword used
**(i) Informal employment**	"informal workers"
**(ii) Risk exposure**	"risk"
**(iii) Psychosocial domain**	"psychosocial"

Conceptual query (databases were queried using each platform's standard interface for keyword search across title, abstract and author-supplied keywords):

```
("informal workers") AND ("risk") AND ("psychosocial")
```

2 Filters applied

- Publication year: 2019 onwards. This lower bound was chosen to capture evidence concurrent with the COVID-19 period and the post-pandemic restructuring of labour markets.
- Language: English. The decision was driven by feasibility and by the prevailing convention of indexing international labour-and-health research in English-language databases. This limitation is acknowledged in Section 5 (Limitations) of the manuscript.
- Document type: scientific journal articles (research articles and reviews).
- Human subjects (where the database supported this filter).

3 Information sources

Four bibliographic databases were queried:

- ScienceDirect (Elsevier)
- Scopus (Elsevier)
- Web of Science Core Collection (Clarivate)
- PubMed (NCBI / MEDLINE)

Each database was queried directly through its native search interface, applying the conceptual query and the filters described in 1 and 2. Reference-list scanning of the included studies was carried out as a complementary step, but did not yield additional eligible primary studies.

4 Pooled records retrieved

Records retrieved from the four databases were pooled and processed jointly through the eligibility steps. Aggregate counts are reported in Table 2 of the main manuscript and reflected in the PRISMA-ScR flow diagram (Figure 1):

PRISMA-ScR stage	Number of records (aggregate)
Identification (across the four databases)	**257**
Screening (after de-duplication and filters)	**193**
Eligibility (full-text assessed)	**18**
Inclusion (primary studies)	**12**

Note. Records were pooled across databases at the identification stage; for this reason, this appendix reports aggregate counts rather than database-by-database counts. The eligibility step excluded 6 records (5 prior systematic reviews / meta-analyses cited only as background literature in the Discussion, and 1 out-of-scope study by Castaldo et al., 2023).

5 Rationale for a focused three-concept strategy

A scoping review is methodologically distinct from a systematic review: its purpose is to map the breadth of available evidence on a topic rather than to provide an exhaustive pooled estimate (Tricco et al., 2018). The Joanna Briggs Institute and Arksey & O'Malley frameworks for scoping reviews accept focused, transparent search strategies provided that the resulting evidence base supports the review's questions.

Here, the three concepts — informal employment, risk, and psychosocial — are the structural elements of the research question (RQ1, RQ1a and RQ1b). Adding broader synonyms (e.g., "informal economy", "precarious work", "job strain") was considered but discarded in order to keep the search focused on the intersection where the three concepts converge, which is the gap identified in the literature. This focused strategy is consistent with the scoping-review purpose of mapping the existing intersection rather than exhaustively recovering every adjacent evidence body.

The limitations of this focused strategy — including the English-only restriction and the absence of grey literature — are acknowledged in Section 5 (Limitations) of the manuscript and are flagged as priorities for future, multilingual evidence syntheses.
